# Consumption of foods with the highest nutritional quality, and the lowest greenhouse gas emissions and price, differs between socio-economic groups in the UK population

**DOI:** 10.1017/S1368980023002355

**Published:** 2023-12

**Authors:** Magaly Aceves-Martins, Ruth L Bates, Leone CA Craig, Neil Chalmers, Graham Horgan, Bram Boskamp, Baukje de Roos

**Affiliations:** 1 The Rowett Institute, University of Aberdeen, Foresterhill, Aberdeen AB25 2ZD, UK; 2 Institute of Applied Health Sciences, University of Aberdeen, Aberdeen, UK; 3 Biomathematics & Statistics Scotland, Rowett Institute, Aberdeen, UK; 4 Biomathematics & Statistics Scotland, The King’s Buildings, Edinburgh, UK

**Keywords:** Diet, Nutrition quality, Greenhouse gas emissions, Cost, Sustainability, UK

## Abstract

**Objective:**

To establish a baseline understanding of whether consuming food with the highest nutritional quality, lowest greenhouse gas emissions (GHGE) and cost differs between different UK demographic and socio-economic population groups.

**Design:**

Multiple linear regression models were fitted to evaluate the relationship between predictor socio-demographic variables in this study (i.e. sex, ethnic group, age, BMI and level of deprivation) and the response variables (i.e. consumption of items considered most nutritious, with a low GHGE and price, as a proportion of total items consumed).

**Setting:**

The UK.

**Participants:**

1374 adult (18–65 years) participants from the National Diet and Nutrition Survey latest waves 9–11 (2016–2017 and 2018–2019).

**Results:**

Based on the total energy consumption in a day, the average diet-based GHGE was significantly higher for participants with a higher BMI. Non-white and most deprived participants spent significantly (*P* < 0·001) less money per total energy consumption. Participants with a BMI between 18·6 and 39·9 kg/m^2^ and those living in the least deprived areas consumed a significantly (*P* < 0·001) higher amount of those items considered the most nutritious, with the lowest GHGE and cost per 100 kcal.

**Conclusions:**

Consumption of food with the highest nutritional quality, lowest GHGE and cost in the UK varies among those with different socio-demographic characteristics, especially the deprivation level of participants. Our analysis endorses the consideration of environmental sustainability and affordability, in addition to the consideration of nutritional quality from a health perspective, to make current dietary guidelines more encompassing and equitable.

The food supply chain is one of the main contributors to greenhouse gas emissions (GHGE), which are currently driving global climate change^(1)^. Rising urbanisation and higher consumption of animal-based products have contributed to increases in GHGE in recent years – it has been estimated that emissions from the food supply chain currently contribute 21–37 % of total GHGE^(2,3)^. Several high-level working groups have highlighted the importance of reducing meat consumption and other animal-origin products to reduce GHGE^(2,4,5)^. Dietary recommendations proposed to reduce GHGE, such as increasing the consumption of plant-origin products and decreasing animal-origin products, also align with current nutritional recommendations developed to reduce mortality^(6–8)^ and the prevalence of major non-communicable diseases, such as cancer or CVD^(6,9,10)^.

It may be challenging for consumers to achieve a dietary pattern that is both healthy and environmentally sustainable as this would involve a cultural and societal shift, mainly in high-income countries where diets and taste preferences are primarily based on animal products^(11,12)^. Moreover, ‘sustainability’ is a complex and comprehensive concept that includes multiple outcomes of environmental and economic impact^(9)^. For instance, environmental sustainability comprises variables such as GHGE, land use, water use and ecotoxicity^(2)^. However, economic affordability is also essential when adopting healthier and sustainable dietary choices^(5,13,14)^. Therefore, affordability is a crucial determinant of food choice and a central contributor to socio-economic inequalities when considering the healthiness of food and drink choices^(12,14)^.

Evidence from high-income countries suggests that dietary choices are influenced by socio-demographic or anthropometric characteristics such as age, sex, education, living situation, marital status or BMI^(15,16)^. For example, less-educated individuals and those on lower incomes had lower Dietary Approaches to Stop Hypertension scores in the UK, mainly driven by a lower intake of fruit, vegetables, whole grains, nuts, legumes and seeds^(17)^. Another UK study found that less-educated individuals and those on lower incomes consumed less fruit and vegetables, with the importance of costs for fruit and vegetables being a significantly stronger factor for food choice in this group than for those on higher incomes and with higher education levels^(18)^. Previously, a framework was developed to quantify actual diet records for health, affordability and environmental sustainability considering UK food purchase survey data^(19)^. However, such studies have yet to simultaneously consider nutritional quality, environmental impact and cost when addressing the food choices and dietary behaviours of UK participants. Therefore, this study aimed to establish a baseline understanding of whether the current consumption of food items with the highest nutritional quality, lowest GHGE and lowest cost differs between different UK demographic and socio-economic population groups.

## Methods

### Data

We analysed data from the UK National Diet and Nutrition Survey (NDNS) latest waves 9–11, which comprise data gathered between 2016–2017 and 2018–2019^(20)^. The NDNS is an annual rolling cross-sectional survey carried out across the UK on behalf of former Public Health England and the Food Standards Agency. This survey collects data from a UK representative sample, including food consumption, nutrient intake and nutritional status. Respondents completed a daily food diary for four consecutive days, including weekends and weekdays. Only the records of those participants who completed 3 or 4 d were included in this analysis. We excluded those with implausible energy intakes below 500 or above 5000 kcal/d^(21)^.

A computer-assisted personal interview collected information on socio-demographic variables, lifestyle factors, dietary habits and height and weight measurements. Only adult participants (18–65 years old) were considered in our analysis. We used socio-demographic data on age, sex, ethnicity and Index of Multiple Deprivation (IMD). The original variable included for ethnicity five ethnic groups, that is, white, mixed ethnic group, black or black British, Asian or Asian British, and any other group. Since most of the survey population was white (89·9 %), the data were aggregated into two categories: white and non-white individuals. We used the IMD as a proxy of socio-economic status in our analysis, which is the official measure of relative deprivation for small areas in the UK. IMD combines information from seven domains (income, employment, education, health, crime, housing and living environment) to produce an overall measure of deprivation. It generates individual estimations based on household-level weighted data calibrated to household population estimates by region and quintiles. Values were equivalised across different countries in the UK by quintiles to indicate the most deprived (IMD 1) and the least deprived (IMD 5) areas.

As part of the NDNS dataset, BMI was calculated as weight (kg) divided by height squared (m^2^) and was categorised as underweight (<18·5 kg/m^2^), normal weight (18·5–24·9 kg/m^2^), overweight (25–29·9 kg/m^2^), obesity (30·0–39·9 kg/m^2^) and severe obesity (≥40 kg/m^2^).

We used an in-house expanded version of the NDNS nutrient databank (2018–2019, available upon request), which includes compositional data but also nutrient profiles, GHGE and cost for nearly 6000 commonly consumed foods and drinks^(20,22,23)^, to identify the food items and food groups with the highest nutritional quality, lowest GHGE and lowest cost. We categorised each item according to their Eatwell Guide food group^(24)^. Also, we assessed nutritional quality by calculating the Nutrient-Rich Food Index 8.3 (NRF8.3)^(25–27)^ per 100 kcal of the food or drink item; the higher the scores, the better the nutritional quality.

In addition, we identified GHGE values for 153 individual foods and dishes, expressed as gCO2-equivalents (CO2e), from open-access sources^(28)^, where possible, from studies using complete cradle-to-grave life cycle analysis^(29)^ following the international PAS 2050 standard^(30)^. For all other food and drink items in the NDNS nutrient databank, three nutrition scientists discussed and imputed reasonable substitute data based on the products’ food type, food group and compositional similarity. Each product’s costs (in GBP) were retrieved up to October 2021 (retail prices were used without adjusting for inflation) using the Shelf Scraper search engine^(31)^(no longer available, but https://www.trolley.co.uk/ may be used instead). Considering that Shelf Scraper did not consider prices at discounters like LIDL or ALDI, the lowest price among leading UK supermarkets (Tesco, ASDA, Sainsbury’s and Morrison’s) was used. For GHGE and cost, data were retrieved for 100 g of product and then estimated per 100 kcal basis.

### Calculation of score

Following the method described by Masset *et al.* ^(32)^, a combined score based on nutritional quality, GHGE and cost for each food or drink in the nutrient databank was developed based on the overall medians for each indicator^(22,23)^. The scoring system ranged from 0 to 3, with each food and/or drink scoring 1 point if the NRF8.3 index score was above the median, 1 point if its GHGE were under the median and 1 point if its cost was under the median. Those items with the highest score (i.e. 3) represented the food items with the highest nutritional quality and lowest GHGE and price per 100 kcal^(22,23)^. This score was estimated for all the items included in the NDNS nutrient databank, regardless of the Eatwell food group they were grouped in.

### Analysis

Overall, demographic, socio-economic and BMI variables were tabulated and presented as counts and percentages. Differences among categorical variables were tested using Chi-square tests, and significance was established at a *P*-value of <0·05. Analysis was done based on each individual’s consumption expressed as total kcal/per day. We then calculated total NRF8.3, GHGE and cost based on each individual’s consumption. These values were averaged per day per person and compared between socio-demographic categories. Statistical differences were tested through one-way ANOVA, and significance was established at a *P*-value of <0·05. In addition, the indicators NRF8.3, GHGE and cost were plotted against consumption (total kcal/d) and analysed for each food group to estimate the contribution of each indicator to the total kcal consumed in a day.

Multiple linear regression models were fitted to evaluate the relationship between predictor socio-demographic variables in this study (i.e. sex, ethnic group, age, BMI and IMD) and the response variable (i.e. consumption of items considered the most nutritious, with a low GHGE and price, as a proportion of total items consumed). The significance of each socio-demographic variable and the response variable were tested to fit the model through simple linear regression models. Also, linearity and residual distributions were visually assessed, and collinearity (through tolerance level to ensure variables were not closely related) was evaluated before modelling the regressions. Finally, *F*-statistics were used to test the significance of each term included in the regression models.

To further assess the differences in total NRF8.3, GHGE and cost per kcal consumed per day, we quantified the individual and between-person variation attributable to different Eatwell Guideline Food Groups. To do so, the variance of NRF8.3, GHGE and cost per kcal consumed per day was calculated. Then, the original values of each item were replaced with the mean of its respective Eatwell food group, and the variances were recalculated. Afterwards, the percentage of variation between the two variances was estimated, and the resultant percentage was attributed to the variation in the Eatwell food group. Next, a *t*-test was used to evaluate the statistical significance between the individual *v*. mean values for NRF8.3, GHGE and cost. In addition, an ANOVA test was used to estimate the statistical significance between the individual and mean values for NRF8.3, GHGE and cost across IMD categories.

Data were visualised through Tableau, and analysis was performed in R software using the libraries ‘*dplyr*’, ‘*psych*’ and ‘*pastecs*’ (for descriptive statistics), and regression models were done using the ‘*lessR*’ library.

## Results

Table 1 summarises the main socio-demographic characteristics of the study population, which included 1374 participants and excluded eighty-six participants based on implausible energy intakes. As expected, the average daily energy intake was significantly lower in females than males. Consequently, diet-based NRF8.3, GHGE and cost per day were significantly lower in females than in males (*P* < 0·001**)** (Table 2). The average diet-based GHGE among the participants differed significantly between body weight categories, with the highest GHGE recorded for those participants with a BMI higher than 25 kg/m^2^. Based on the daily energy consumption, the average food and drink intake cost was significantly lower in the non-white compared with white UK participants (*P* < 0·001). Additionally, the average cost of daily food and drink intake differed significantly (*P* < 0·001) across IMD categories, with the most deprived participants (IMD categories 1 and 2) spending less per total energy consumed in a day (Table 2).

**Table 1:** Demographic characteristics of the study population

Demographic characteristics	UK sample (*n* 1374)	
	*n*	%
Sex		
Females	847	62
Males	527	38
Ethnicity		
White	1222	30
Non-white	152	11
BMI*		
<18·5	13	1
18·6–24·9	476	37
25·0–30·0	479	37
30·1–39·9	264	21
>40	49	4
IMD		
1	274	20
2	297	22
3	249	18
4	268	19
5	286	21
Age		
	Mean	sd
	42·1	12·9

* BMI data are available only for 1281 participants in the UK sample.

IMD – Index of Multiple Deprivation is the official measure of relative deprivation for small areas in the UK, 1 being the most and 5 being the least deprived. The participants’ proportions differed significantly (*P* < 0·05) according to their sex and ethnicity.

**Table 2:** Average energy intake, NRF8.3, GHGE and cost of food and drink consumption per day

Socio-demographic categories characteristics	Total energy consumption per day		Total NRF8.3		Total GHGE (gCO2e)		Total cost (GBP)		Total number of food items scoring ‘three’†	
	Mean	sd	Mean	sd	Mean	sd	Mean	sd	Mean	sd
Overall	2216	962·9	6341·4	2724·4	5428·4	2648·0	8·55	5·8	1·3	0·8
Sex										
Male	2735·3	977·9**	7638·1	2780·3***	6579·2	2825·7***	10·1	6·3***	1·3	0·8
Female	1894·4	799·1***	5534·6	2353·8***	4712·4	2253·3***	7·5	5·2***	1·3	0·8
Ethnicity										
White	2227·0	970·2	6360·9	2730·6	5427.	2629·5	8·7	5·8***	1·3	0·8
Non-white	2138	909·8	6167·3	2696·8	5458·7	2817·3	6·6	4·6***	1·2	0·7
BMI										
<18·5	2308	1093·0	5886·0	3193·6	5216·3	2545·4**	8·2	5·1	0·9	0·3*
18·6–24·9	2106·8	924·0	6052·1	2575·2	5092·5	2571·3**	8·1	5·3	1·4	0·9*
25·0–30·0	2290·5	981·4	6603·0	2799·1	5669·8	2602·3**	8·9	6·0	1·3	0·8*
30·1–39·9	2280·9	956·9	6516·9	2591·8	5634·1	2741·5**	8·9	6·2	1·3	0·8*
>40	2107·95	1030·9	5750·3	2835·7	5472·0	3217·1**	8·0	5·5	1·1	0·63*
IMD										
1	2196	981·2	6096·4	2692·4	5272·6	2605·7	7·56	5·3***	1·2	0·7**
2	2235·6	931·4	6413·8	2735·0	5500·6	2725·0	7·97	5·2***	1·2	0·7**
3	2341·9	1043·4	6764·5	3120·2	5757·6	2989·3	9·04	6·2***	1·4	0·9**
4	2164·0	921·7	6231·2	2464·5	5285·0	2360·6	9·0	6·0***	1·4	1·0**
5	2157·5	937·8	6235·9	2576·8	5350·6	2533·5	9·2	5·9***	1·3	0·8**

The statistical significance was defined as:

* 0·01.

† Those items with the highest score (i.e. 3) represented the food items with the highest nutritional quality and lowest GHGE and price/100 kcal.

** 0·001.

*** <0·001.

Data represent the mean (sd).

Data of eighty-six participants were removed since the average energy consumption/d was lower than 500 kcal or higher than 5000 kcal.

The total analysis sample consisted of 1374 participants.

Total NRF8.3, GHGE and cost values are based on total daily energy consumption.

IMD – Index of Multiple Deprivation is the official measure of relative deprivation for small areas in the UK, 1 being the most and 5 being the least deprived.

IMD was standardised across countries of the UK; BMI was estimated as weight (kg) divided by height squared (m^2^), and it was categorised as underweight (<18·5 kg/m^2^), normal weight (18·5–24·9 kg/m^2^), overweight (25–29·9 kg/m^2^), obesity (30·0–39·9 kg/m^2^) and severe obesity (≥40 kg/m^2^).

Sex and ethnicity significance were evaluated with a *t*-test, and the rest of the variables was analysed with one-way ANOVA within demographic categories.

In total, 577 (out of 4910) food items scored 3 in our analysis (e.g. representing foods with the highest nutritional quality, lowest GHGE and lowest cost per 100 kcal). A list with the distribution of these items according to the Eatwell Guide and some examples can be consulted in Aceves-Martins *et al*.^(22)^. The total amount of items scoring 3 (representing foods with the highest nutritional quality, lowest GHGE and lowest cost per 100 kcal) that were consumed in a day was significantly (*P* < 0·01) different between BMI categories, with the lowest number being consumed in those having a BMI between ≤18·5 kg/m^2^. Also, the total number of food items scoring a 3 (representing foods with the highest nutritional quality, lowest GHGE and lowest cost per 100 kcal) was significantly (*P* < 0·001) different between IMD categories, with those living in the least deprived areas, for example, IMD categories 3, 4 or 5, consuming a higher proportion of these products (Table 2).

**Fig. 1:**
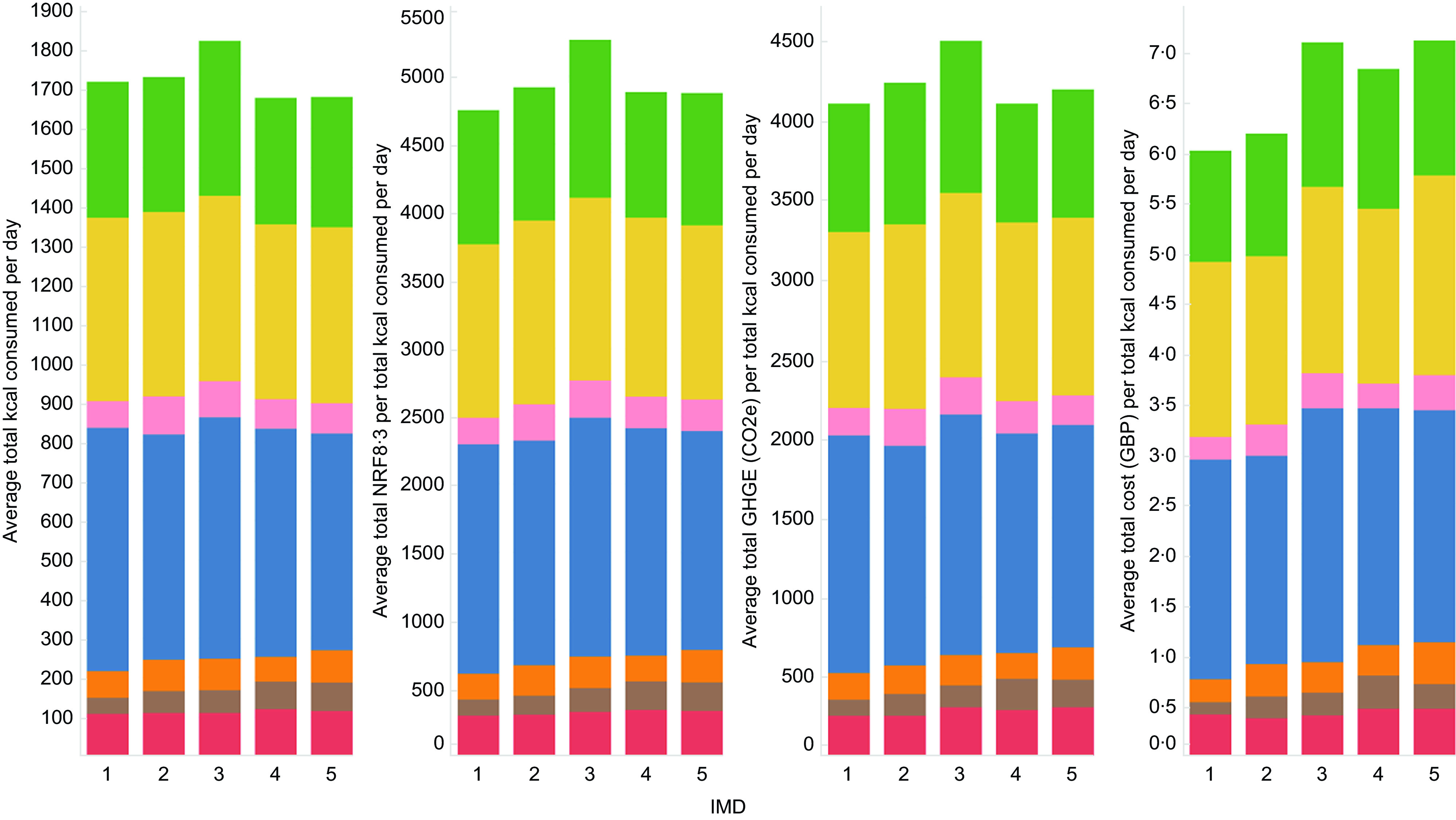
The average kcal consumed per day and total NRF8.3, GHGE and cost per total kcal consumed in a day, for each of the Eatwell Guide food groups across IMD categories. Food groups 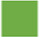 fruits and vegetables; 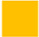 potatoes, bread, pasta, rice and other starchy carbohydrates; 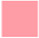 beans, pulses, fish, eggs, meat and other proteins; 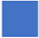 dairy and alternatives; 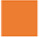 oils and spreads; 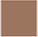 drinks; 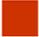 miscellaneous items that should be eaten less often and in small amounts. Kcal, kilocalories; NRF8.3, Nutrient-Rich Food Index 8.3; GHGE, greenhouse gas emissions expressed as gCO2-equivalents (CO2e); GBP, Great British Pound; IMD, Index of Multiple Deprivation with IMD 1 being the most deprived and IMD 5 being the least deprived areas

The average kcal consumed per day and total NRF8.3, GHGE and cost per total kcal consumed in a day for each of the Eatwell Guide food groups and across IMD categories are presented in Fig. 1. The highest proportion of the kcals consumed in a day came from the Eatwell food group dairy and alternatives, followed by potatoes, bread, rice, pasta and other starchy carbohydrates and then from fruit and vegetables. As a result, we observed higher values for nutritional quality and GHGE in these food groups.

Only sex, ethnicity and IMD were suitable (i.e. significant at simple linear regressions) to include in the multiple linear regression model. These socio-demographic variables were significantly related to the total number of items scoring 3 in a day (Table 3). Of these, only sex and IMD indicated a unique significant variance, with the total number of items scoring 3 consumed in a day (*P* = 0·007 and *P* = 0·003, respectively), even when controlling for other variables. Overall, collectively sex, ethnicity and IMD could explain a small but significant variability (1·4 %, adjusted *R* ^2^ = 0·14, *P*-value<0·0001) in the total number of food items scoring a 3, having the highest nutritional quality and lowest GHGE and cost consumed in a day. Furthermore, when analysing each indicator (i.e. NRF 8.3, GHGE and cost) separately, we found that sex, ethnicity and IMD also explained a small but significant variability for each indicator (Appendix 1, see online Supplemental Tables (a–d)).

**Table 3:** Multiple linear regression model to evaluate the relationship between sex, ethnicity and IMD and consumption of food products with the highest nutritional quality and the lowest GHGE and price

Parameter	Estimate	SE	*t*-value	*P*
Intercept	0·86447558	0·08 894 743	9·719	<0·001
Sex (female)	−0·08 591 524	0·03 207 114	−2·679	0·007
Ethnicity (non-white)	−0·09 444 639	0·05 021 596	−1·881	0·060
IMD	0·03 236 322	0·01 105 476	2·928	0·003

The statistical significance was defined as: *P*-value < 0·01; *P*-value < 0·001; *P*-value < 0.0001.

Model fit: SD of the average number of items scoring 3: 0·58179863; SD of residuals: 0·57655339 for 1367 df; 95 % range of residual variation: 2·26205060 = 2 * (1·962 × 0·57655339); *R*^2^: 0·016; adjusted *R*^2^: 0·014; *F*-statistic: 7·509; *P*-value: 0·001.

We observed significant (*P* < 0·001) between-person variation in individual *v*. median values for NRF8.3, GHGE and cost across all Eatwell Guide food groups. The variation for the three indicators was noticeably higher in the fruits and vegetables food group and lowest in the dairy and alternative food group. Between-person variation in individual *v*. median values for NRF8.3, GHGE and cost also differed significantly across IMD categories for each food group (Table 4).

**Table 4:** NRF8.3, GHGE and cost between-person variation attributable to different food groups and IMD

Food group	Indicator	Total variation (%)	Total variation (%) per IMD				
			1	2	3	4	5
Fruit and vegetables	Cost***	329·1***	308·6***	390·9***	305·6***	377·5***	270·3***
	NRF8·3***	433·2***	394·4***	573·0***	387·9***	456·3***	363·2***
	GHGE***	167·9***	119·5***	199·3***	158·6***	189·3***	165·1***
Potatoes, bread, rice, pasta and other starchy carbohydrates	Cost***	3·3***	1·3***	1·8***	2·9***	5·0***	7·2***
	NRF8·3***	5·0***	4·6***	6·7***	2·9***	3·3***	7·7***
	GHGE***	2·9***	0·3***	4·0***	1·6***	3·3***	5·4***
Beans, pulses, fish, eggs, meat and other proteins	Cost*	20·4**	17·1***	14·6	22·7*	25·7*	23·3
	NRF8·3**	11·3***	8·7	5·1	12·9***	17·5***	14·8***
	GHGE*	68·8***	71·4***	62·8***	68·6***	66·8***	74·6***
Dairy and alternatives	Cost*	0·4***	0·8***	0·8***	1·4***	0·1***	0·6***
	NRF8·3**	1·6***	0·8***	3·8***	0·7***	1·2***	1·2***
	GHGE*	0·5***	0·2***	1·5***	0·6***	0·5***	1·5***
Oils and spreads	Cost***	108·6***	95·1***	106·5***	75·2***	94·8***	158·4***
	NRF8·3***	165·2***	149·1***	175·0***	117·7***	142·3***	226·7***
	GHGE**	60·9***	42·2***	64·2***	52·4***	40·5***	85·9***
Drinks	Cost*	44·1***	38·8***	52·4***	44·8***	39·8***	42·3***
	NRF8·3*	21·7***	18·7***	25·4***	19·9***	23·8***	20·7***
	GHGE*	9·3***	6·5***	9·7***	8·3***	16·6***	6·1***
Miscellaneous items that should be eaten less often and in small amounts	Cost***	8·9***	4·8***	19·4***	4·2***	17·7***	14·0***
	NRF8·3***	5·5***	16·8***	2·7***	20·2***	3·1***	0·1***
	GHGE***	9·0***	3·4***	11·9***	2·1***	2·1***	17·1***

NRF8.3, Nutrient-Rich Food Index 8.3; GHGE, greenhouse gas emissions expressed as gCO2-equivalents (CO2e); GBP: Great British Pound; IMD, Index of Multiple Deprivation, with IMD 1 being the most deprived and IMD 5 being the least deprived areas.

The statistically significant difference in the total variability was

* 0·01 compared with the original values.

** 0·001.

*** <0·001.

Data from eighty-six participants were removed since the average daily energy consumption was lower than 500 kcal or higher than 5000 kcal.

The total analysis sample comprised 1374 participants.

## Discussion

This study aimed to establish a baseline understanding of whether the UK’s current consumption of food items with the highest nutritional quality, lowest GHGE and lowest cost varies among those with different socio-demographic characteristics. Based on the total energy consumption in a day, the highest food-related GHGE was recorded for participants with a BMI higher than 25 kg/m^2^. We also found that the average cost of daily energy intake was significantly lower in non-white compared with white UK participants. Moreover, the most deprived participants (IMD categories 1 and 2) spent significantly less money per total energy consumed per day than the least deprived participants, and those living in the least deprived areas (IMD categories 3, 4 or 5) consumed more products considered the most nutritious, environmentally friendly and cheap. Overall, sex, ethnicity and IMD explained a small but significant variability in the total number of foods consumed considered to be the most nutritious, with the lowest GHGE and cost. Furthermore, we found significant between-person variation in NRF8.3, GHGE and cost of food groups across IMD categories, with the highest variation recorded for the food group of fruits and vegetables and the lowest variation recorded for the food group diary and alternatives.

Several studies have reported differences and inequalities in dietary patterns across socio-economic groups in the UK^(15,17,18,33,34)^, establishing that income levels, deprivation or education are associated with specific dietary consumption patterns^(13,18,35,36)^. Overall, most studies agree that those with low socio-economic status, less education and no employment or lower employment qualifications struggle to achieve patterns that conform with healthy dietary guidelines^(13,18,35,36)^. Similarly, our results showed that those most deprived consumed significantly fewer food items with the highest nutritional quality and lowest GHGE and cost. We also found that non-white participants, and those living in the most deprived areas, spent less per daily energy consumed than white participants or those living in areas with a low level of deprivation. Thus, our food-based analysis aligns with previous food or diet-based studies that have reported that adherence to healthier diets is associated with higher dietary costs in the UK^(19,34,37,38)^. However, such adherence can vary according to socio-economic status, sex, age, work type and ethnicity^(34,37,38)^.

We found that overall sex, ethnicity and the level of deprivation explained a small but significant variability in the total number of foods consumed considered the most nutritious, with the lowest GHGE and cost, with only sex and the level of deprivation indicating a unique variance. Unlike most current dietary analyses that report diet differences across socio-demographic and economic characteristics^(12,18,34,37,38)^, we simultaneously consider three relevant indicators (i.e. nutritional quality, GHGE and cost) within the same metric. A previous study led by Masset *et al.* ^(32)^ conducted a similar analysis in a French population study and also found differences between females and males regarding the consumption of the most sustainable items, as we did. However, this was the only characteristics of the participants that were included in that analysis. Considering the major global public health (i.e. obesity) and environmental (i.e. climate change) challenges we face, simultaneously considering such indicators is imperative to achieve healthier and more sustainable dietary choices across all populations, regardless of socio-demographic characteristics. A recent study^(35)^ modelled the dietary changes required to shift patterns that meet dietary recommendations towards a healthier, more sustainable (lower GHGE) and affordable diet across different income groups. This study^(35)^ concluded that changing dietary patterns could be achieved within current household food budgets by altering the amounts of specific foods consumed (e.g. increasing fruit, vegetables and starchy foods, while reducing animal products and high-fat/high-sugar foods) rather than eliminating foods.

From a consumer’s perspective, distinguishing healthy, sustainable and fair food prices based on individual food products instead of whole diets could promote better shopping choices^(32,39)^. Our analysis estimated between-participant variation for each indicator (i.e. nutritional quality, GHGE and cost). As a result, we observed significant between-person variation in individual *v*. median values for NRF8.3, GHGE and cost across all food groups, which differed significantly between IMD categories. This highlights the opportunities for personalised approaches to optimise the intake of healthier, greener and more affordable products, where simple substitutions within food subgroups or ‘food swaps’ could effectively improve the nutritional adequacy of the diets^(40–42)^.

Although we found significant between-participant variation in NRF8.3, GHGE and/or cost between food groups, this was notably higher for the fruits and vegetables food group. This high between-participant variation in fruit and vegetable intake might reflect the considerable variation in nutritional quality, environmental markers and cost in this food group, especially when considering the difference in the amounts consumed per individual. Previous UK studies^(15,17,18,33,34)^ have reported that the most disadvantaged populations struggle to achieve the recommended amount of five fruits and vegetables per day, mainly linked to affordability. Fruits and vegetables are pivotal for a healthy and sustainable diet^(24)^. Unfortunately, with the information from NDNS, it is impossible to estimate the proportion of imported or locally grown and cultivated foods. However, the UK’s average consumption of fruits and vegetables is less than 300 g/d^(43)^. Furthermore, only 7 % of the fruits we consume are produced in the UK, with the rest being imported, primarily (70 %) from outside of Europe, implying an environmental impact^(43)^ and affecting the cost.

We analysed data based on the energy consumed (kcal/d) per participant rather than the weight consumed (g). Therefore, the NRF used in our analysis was estimated per kcal. However, some studies have analysed data per serving size^(25)^ or 100 g^(26)^. When considering the nutritional quality estimated through NRF, it has been acknowledged that foods that benefited the most from the 100-kcal calculation were low-energy-dense such as vegetables. On the other hand, foods that benefited more from the 100-g calculation were energy-dense foods such as nuts and seeds or fortified cereals. Indeed, different studies have highlighted that the metric used to measure nutritional quality, environmental impact or cost of food items can affect which foods are considered more nutritious, environmentally friendly and cheaper^(25,26,44)^.

Some strengths of our work include simultaneously considering relevant nutritional quality indicators, GHGE as an essential marker of environmental sustainability and the cost of an extensive range of UK food items and applying these to dietary records from the NDNS data, which provides high-quality dietary intake data in a UK nationally representative sample. However, there are some limitations linked to the use of these data. For instance, the NDNS survey relies on self-reported food intake, which might not be accurate because of participants’ (un)conscious misreporting, as per BMI categories^(45,46)^. In fact, we observed a lower energy intake for the highest BMI category, which may show under-reporting of food intake, a well-known issue, especially in those with obesity^(47)^. Consequently, this would have affected this group’s NRF8.3, GHGE and cost results. Second, the survey includes mainly white participants, thereby overlooking the dietary patterns of ethnic minorities. Third, although it considered participants from all deprivation levels, highly vulnerable participants (i.e. homeless or migrants who are not English speakers) were least likely to be included in this survey^(33,48)^. Also, although IMD is a comprehensive index, it is based on the postcode of participants. By using this index, some of the granularity of the data related to different socio-economic factors might be missed. Moreover, although IMD was standardised for this study’s analysis, this index is slightly different across the UK. Fourth, because of the fluctuation of food prices and data collected in 2021, we limited our analysis to the NDNS latest waves (2016–2017 and 2018–2019), omitting previous valuable data. In addition, by using the prices retrieved in 2021, we realise that data on food costs may only partially align with those when the survey was conducted. Still, the current picture might be different considering the most recent cost of living crisis with a clear rising food cost. Finally, we used GHGE as an environmental marker. Although GHGE strongly correlates with water eutrophication and air acidification^(32)^, this might not reflect other relevant environmental indicators in food products, such as water blueprint^(49)^ or ecosystem biodiversity losses^(50)^. Hence, future research needs to consider integrating other environmental impact measurements, such as land or water use, into one metric for environmental sustainability.

Public health policies and dietary guidelines should consider the impact of dietary choices in terms of nutritional quality and environmental and economic impact. Here, we provide evidence that the UK’s consumption of food items with the highest nutritional quality, lowest GHGE and lowest cost varies among those with different socio-demographic characteristics, especially the deprivation level of participants. Moreover, this analysis provides new opportunities to identify ‘food swaps’ that allow individuals to make their diets healthier and greener while not compromising on price. Such dietary improvement through ‘food swaps’ should also consider the level of processing as well as the composition of foods (e.g. to reduce discretionary foods consumption). Future research needs to consider equity regarding how achievable dietary guidelines are and how affordability and accessibility are crucial for consumption. As shown by the results presented in our analysis, special attention needs to be paid to those living in the most deprived areas, as they might struggle more to meet such guidelines. This is key to improving health outcomes across populations and achieving the climate change targets to prevent global warming.

## Supporting information

Aceves-Martins et al. supplementary material Aceves-Martins et al. supplementary material

## Supplementary material

For supplementary material accompanying this paper visit https://doi.org/10.1017/S1368980023002355
