# Supplementary material for: Consumption of foods with the highest nutritional quality, and the lowest greenhouse gas emissions and price, differs between socio-economic groups in the UK population
Source: Public Health Nutr. 2023 Oct 31;26(12):3370–8. doi: 10.1017/S1368980023002355 (PMC10755419; doi:10.1017/S1368980023002355)
Supplement: Aceves-Martins et al. supplementary material [file S1368980023002355sup001.docx]

**Appendix 1. Regression models for secondary outcomes (i.e., models for each score component: NRF8.3, GHGE and Cost) and Kcal consumed in a day.**

**Supplementary Table A.** Kcal consumed per day

| **Parameter** | **Estimate** | **Std. Error** | **t-value** | **P-value** |
| --- | --- | --- | --- | --- |
| Intercept | 3803.96329 | 134.47899 | 28.287 | 0.000 |
| Sex (Female) | -847.34606 | 48.48812 | -17.475 | 0.000 |
| Ethnicity (Non-white) | -98.59446 | 75.92116 | -1.299 | 0.194 |
| IMD | -36.10318 | 16.71361 | -2.160 | 0.031 |

Model Fit

Standard deviation of kcal consumed on a day: 962.90622; Standard deviation of residuals: 871.68700 for 1367 degrees of freedom; 95% range of residual variation: 3,419.97830 = 2 * (1.962 * 871.68700); R^2^: 0.184; Adjusted R^2^: 0.182; F-statistic: 102.659; p-value: 0.000

**Supplementary Table B.** Total NRF8.3 index per total kcal consumed per day.

| **Parameter** | **Estimate** | **Std. Error** | **t-value** | **P-value** |
| --- | --- | --- | --- | --- |
| Intercept | 10086.2307 | 389.9443 | 25.866 | 0.000 |
| Sex (Female) | -2115.5919 | 140.5994 | -15.047 | 0.000 |
| Ethnicity (Non-white) | -184.9334 | 220.1461 | -0.840 | 0.401 |
| IMD | -41.1592 | 48.4639 | -0.849 | 0.396 |

Model Fit

Standard deviation of average NRF8.3 of products consumed in a day: 2,724.4211; Standard deviation of residuals: 2,527.6019 for 1367 degrees of freedom; 95% range of residual variation: 9,916.7978 = 2 * (1.962 * 2,527.6019) R^2^: 0.143; Adjusted R^2^: 0.141; F-statistic: 75.736; p-value: 0.000

**Supplementary Table C.** Total GHGE (gCO2e) per total kcal consumed per day.

| **Parameter** | **Estimate** | **Std. Error** | **t-value** | **P-value** |
| --- | --- | --- | --- | --- |
| Intercept | 8556.5167 | 384.3648 | 22.261 | 0.000 |
| Sex (Female) | -1874.2126 | 138.5877 | -13.524 | 0.000 |
| Ethnicity (Non-white) | 33.1609 | 216.9961 | 0.153 | 0.879 |
| IMD | -44.9650 | 47.7705 | -0.941 | 0.347 |

Model Fit

Standard deviation of Average GHGE per kcal consumed in a day: 2,648.0279; Standard deviation of residuals: 2,491.4360 for 1367 degrees of freedom; 95% range of residual variation: 9,774.9044 = 2 * (1.962 * 2,491.4360); R^2^: 0.118 Adjusted R^2^: 0.116; F-statistic: 60.975; p-value: 0.000

**Supplementary Table D.** Total Cost (GBP) per total kcal consumed per day.

| **Parameter** | **Estimate** | **Std. Error** | **t-value** | **P-value** |
| --- | --- | --- | --- | --- |
| Intercept | 13.76245688 | 0.86649191 | 15.883 | 0.000 |
| Sex (Female) | -2.55291984 | 0.31242477 | -8.171 | 0.000 |
| Ethnicity (Non-white) | -1.83558565 | 0.48918474 | -3.752 | 0.000 |
| IMD | 0.32039178 | 0.10769125 | 2.975 | 0.003 |

Model Fit

Standard deviation of cost per kcal consumed in a day: 5.80357294; Standard deviation of residuals: 5.61656324 for 1367 degrees of freedom; 95% range of residual variation: 22.03603410 = 2 * (1.962 * 5.61656324); R^2^: 0.067; Adjusted R^2^: 0.065; F-statistic: 32.648; p-value: 0.000
